# Association of Sodium-Glucose Cotransporter-2 Inhibitors With Atrial High-Rate Episodes in Patients With Cardiac Implantable Electronic Devices

**DOI:** 10.7759/cureus.105732

**Published:** 2026-03-23

**Authors:** Wichid Thirakraisri, Thipsukhon Sathapanasiri, Pariwat Pengkaew, Surachat Jaroonpipatkul

**Affiliations:** 1 Division of Cardiology, Rajavithi Hospital, College of Medicine, Rangsit University, Bangkok, THA; 2 Rheumatic Unit, Department of Internal Medicine, Bangkok Hospital Headquarter, Bangkok, THA; 3 Bangkok Health Research Center, Bangkok Hospital Headquarter, Bangkok, THA

**Keywords:** atrial fibrillation, atrial high-rate episodes, cardiac implantable electronic device, major adverse cardiovascular events, sodium-glucose cotransporter-2 inhibitors

## Abstract

Background and objective

Atrial high-rate episodes (AHREs) are subclinical atrial tachyarrhythmias detected by cardiac implantable electronic devices (CIEDs). Identifying strategies to reduce AHRE occurrence is clinically relevant. Sodium-glucose cotransporter-2 inhibitors (SGLT2i) are commonly prescribed for cardiometabolic conditions, and their potential role in subclinical atrial arrhythmias remains to be clarified. The objective of this study is to compare the incidence of AHREs and major adverse cardiovascular events (MACE) between SGLT2i users and nonusers among CIED patients.

Methods

This retrospective cohort study included CIED recipients from January 2020 to December 2024. Patients were categorized into two groups based on SGLT2i exposure. Clinical characteristics, device parameters, and AHRE data were collected and analyzed. MACE were also recorded. Multiple imputation was applied for missing left ventricular ejection fraction and left atrial volume index data. Propensity score overlap weighting was used to balance baseline covariates. Cox proportional hazards models estimated HRs for AHREs and MACE.

Results

A total of 123 patients with CIEDs were included, with a mean age of 73.3 ± 13.1 years, and 46.3% were male. Of these, 15 patients received SGLT2i. Overlap propensity score weighting was applied. During follow-up, AHREs occurred in three patients (SGLT2i group) and 54 patients (non-SGLT2i group), corresponding to weighted incidence rates of 40.8 and 1304.9 per 1,000 person-years, respectively. SGLT2i use was associated with a significantly lower risk of AHRE occurrence (HR 0.32, 95% CI 0.10-0.98, p = 0.03). For MACE, three events were observed in the SGLT2i group and 20 in the non-SGLT2i group, yielding a weighted HR of 1.10 (95% CI 0.25-4.96, p = 0.10). Kaplan-Meier survival analysis demonstrated significantly higher AHRE-free survival among patients receiving SGLT2i (log-rank p = 0.03), whereas MACE-free survival did not differ significantly between the groups (p = 0.10). The duration of the longest single AHRE episode was numerically shorter among SGLT2i users, although the difference was not statistically significant.

Conclusions

SGLT2i use was associated with a significantly lower risk of AHRE occurrence among CIED recipients, suggesting a potential reduction in subclinical atrial arrhythmia burden. However, no significant difference in MACE was observed. These findings highlight a possible antiarrhythmic benefit beyond established cardiovascular effects. Larger prospective studies are warranted to confirm these results.

## Introduction

Atrial fibrillation (AF) affects approximately 33.5 million adults globally and is associated with increased risks of stroke, heart failure, and mortality [1]. In Thailand, AF prevalence ranges from 0.4% in individuals aged ≥30 years to 1.9% in those aged ≥65 years [2]. Atrial high-rate episodes (AHREs) represent rapid atrial arrhythmias detected through continuous monitoring by cardiac implantable electronic devices (CIEDs), including pacemakers, implantable cardioverter-defibrillators (ICDs), and cardiac resynchronization therapy (CRT) devices [3,4]. AHREs occur in 10-30% of elderly patients without a prior history of AF and significantly increase stroke risk compared with patients without AHREs [3,4]. Furthermore, patients with AHREs demonstrate a 4.45-fold higher likelihood of developing clinical AF [5].

The pathophysiology of AHREs involves complex mechanisms, including atrial remodeling characterized by electrical changes, such as reduced refractory period and increased automaticity, and structural alterations, including atrial fibrosis and dilatation [6-8]. Additional contributing factors include inflammation and oxidative stress [9,10], autonomic nervous system dysfunction [11,12], elevated atrial pressure and stretch [13,14], and comorbid conditions such as diabetes mellitus, hypertension, and heart failure [15].

Sodium-glucose cotransporter-2 inhibitors (SGLT2i), initially developed as glucose-lowering agents for type 2 diabetes, have demonstrated remarkable cardiovascular benefits. Clinical trials have shown that SGLT2i reduce heart failure hospitalizations by 28% and cardiovascular mortality by 23% in patients with type 2 diabetes and heart failure [16]. Recent evidence suggests that SGLT2i may reduce AF incidence in patients with type 2 diabetes and heart failure [17], but their effect on subclinical AHREs remains inadequately investigated.

The mechanisms by which SGLT2i might prevent AHREs potentially involve reduction of atrial remodeling through decreased cardiac preload and afterload [13,14], amelioration of systemic inflammation and oxidative stress [18], and improvement in autonomic regulation, potentially through attenuation of sympathetic overactivity and favorable hemodynamic effects [19,20]. Given the clinical significance of AHREs as precursors to clinical AF and thromboembolic events, understanding the potential protective effects of SGLT2i could have important therapeutic implications. This study aimed to investigate the association between SGLT2i use and AHRE incidence in patients with CIEDs.

## Materials and methods

This retrospective cohort study was conducted at Rajavithi Hospital, Bangkok, Thailand, from January 2020 to December 2024. The study protocol was approved by the Institutional Review Board of Rajavithi Hospital (approval 68106) in accordance with the Declaration of Helsinki.

We included adult patients (≥18 years) who underwent CIED implantation and had device follow-up at Rajavithi Hospital during the study period. Retrospective data collection was performed from the time of device implantation. CIEDs included permanent pacemakers, ICDs, and CRT devices. Patients were excluded if they had persistent or permanent AF at baseline or implantation with single-chamber ICDs.

Patients were categorized into two groups based on SGLT2i exposure: SGLT2i users were defined as patients receiving any SGLT2i, while nonusers never received SGLT2i therapy. The decision to prescribe SGLT2i was made by treating physicians according to clinical indications and was not influenced by this research.

Data were extracted from electronic medical records and CIED interrogation reports. Baseline characteristics included demographic data (age, sex, and BMI), comorbidities (diabetes mellitus, hypertension, obstructive sleep apnea, chronic obstructive pulmonary disease, obesity, chronic kidney disease, hyper- or hypothyroid disease, and heart failure), clinical parameters (systolic and diastolic blood pressure and CHA₂DS₂-VA score), laboratory parameters (estimated glomerular filtration rate calculated using the CKD-EPI equation), echocardiographic parameters (left ventricular ejection fraction (LVEF) and left atrial volume index (LAVI)), CIED-related variables (device type, lead position, and atrial and ventricular pacing percentages), and medication use.

The primary outcome was the occurrence of AHREs, defined according to the current European Society of Cardiology guidelines as atrial arrhythmias with atrial rate >175 beats per minute lasting ≥5 minutes, as detected by CIED interrogation [21]. Secondary outcomes included AHRE duration, defined as the longest single AHRE episode (in hours) recorded during device follow-up, and major adverse cardiovascular events (MACE), defined as a composite of myocardial infarction (MI), stroke, heart failure hospitalization, and cardiovascular death.

Missing data in LVEF and LAVI were addressed using multiple imputation via chained equations, generating 20 imputed datasets. Baseline characteristics were summarized using descriptive statistics. Continuous variables with a normal distribution were expressed as mean ± SD, while non-normally distributed variables were presented as median with IQR. Categorical variables were reported as frequencies and percentages.

To reduce confounding, propensity score overlap weighting was applied within each imputed dataset to achieve covariate balance across treatment groups. Standardized mean differences (SMDs) were used to assess balance before and after weighting, with values <0.1 considered indicative of adequate balance. After confirming the proportional hazards assumption using log-log survival plots and Schoenfeld residuals, weighted Cox proportional hazards models were conducted to examine the association between SGLT2i use and AHREs and MACE.

We also compared AHRE duration between groups using weighted analyses. All treatment effect estimates were derived within each imputed dataset and pooled using Rubin’s rules to obtain final HRs with 95% CIs. All analyses were conducted using STATA 19 (StataCorp, College Station, TX, USA), with a two-sided p-value <0.05 considered statistically significant.

## Results

A total of 123 patients with CIEDs were included in the study, comprising 15 SGLT2i users (12.2%) and 108 nonusers (87.8%). Before propensity score weighting, substantial imbalances existed between groups. SGLT2i users were younger (mean age 69.6 ± 17.35 vs 73.81 ± 12.45 years, SMD = 0.530), more likely to be male (66.67% vs 43.53%, SMD = 0.469), had higher BMI (25.60 ± 5.12 vs 24.34 ± 4.46 kg/m², SMD = 0.262), higher prevalence of diabetes mellitus (53.33% vs 33.33%, SMD = 0.180) and heart failure (40.00% vs 3.70%), and lower mean LVEF (48.16 ± 15.70% vs 61.13 ± 16.12%, SMD = 0.846). After overlap weighting, baseline covariate balance was substantially improved compared with the unweighted cohort (Table 1).

**Table 1:** Baseline characteristics before and after propensity score weighting This table summarizes the demographic and clinical characteristics of patients receiving versus not receiving SGLT2i before and after propensity score overlap weighting. Data are presented as mean ± SD, median (IQR), or n (%). CKD, chronic kidney disease; COPD, chronic obstructive pulmonary disease; DM, diabetes mellitus; HTN, hypertension; LAVI, left atrial volume index; LVEF, left ventricular ejection fraction; OSA, obstructive sleep apnea; SGLT2i, sodium-glucose cotransporter-2 inhibitor; SMD, standardized mean difference

Characteristics	Before weighting			After weighting		
	SGLT2i (n = 15)	Non-SGLT2i (n = 108)	SMD	SGLT2i (n = 15)	Non-SGLT2i (n = 108)	SMD
Age (years) (mean ± SD)	69.6 ± 17.35	73.81 ± 12.45	0.530	70.52 ± 17.05	70.42 ± 12.78	0.039
Sex			0.469			0.028
Male (n, %)	10 (66.67%)	47 (43.53%)		9 (62.39%)	69 (63.49%)	
Female (n, %)	5 (33.33%)	61 (56.48%)		6 (37.61%)	39 (36.51%)	
BMI (kg/m²) (mean ± SD)	25.60 ± 5.12	24.34 ± 4.46	0.262	25.57 ± 5.19	25.60 ± 4.97	0.108
Comorbidities			0.180			0.187
DM (n, %)	8 (53.33%)	36 (33.33%)		8 (54.36%)	49 (45.30%)	
HTN (n, %)	8 (53.33%)	67 (62.04%)		8 (54.36%)	75 (69.07%)	
OSA (n, %)	0 (0.00%)	1 (0.93%)		0 (0.00%)	1 (0.61%)	
COPD (n, %)	0 (0.00%)	3 (2.78%)		0 (0.00%)	2 (2.05%)	
Obesity (n, %)	1 (6.67%)	1 (0.93%)		1 (7.06%)	2 (1.55%)	
CKD (n, %)	3 (20.00%)	24 (22.22%)		3 (19.46%)	24 (22.66%)	
Thyroid disease (n, %)	0 (0.00%)	10 (9.26%)		0 (0.00%)	7 (6.16%)	
Heart failure (n, %)	6 (40.00%)	4 (3.70%)		6 (37.48%)	10 (9.01%)	
Echocardiographic parameters						
LVEF (mean ± SD)	48.16 ± 15.70	61.13 ± 16.12	0.846	50.30 ± 15.10	49.69 ± 19.86	<0.001
LAVI (mean ± SD)	35.20 ± 15.30	36.79 ± 23.35	0.08	34.68 ± 14.68	34.66 ± 18.88	<0.001
CHA₂DS₂-VA (median, IQR)	2 (2, 4)	3 (2, 4)	0.127	2 (2, 4)	3 (2, 4)	0.227

The weighted groups were more comparable for age (70.52 ± 17.05 vs 70.42 ± 12.78 years, SMD = 0.039), sex distribution (62.39% vs 63.49% male, SMD = 0.028), BMI (25.57 ± 5.19 vs 25.60 ± 4.97 kg/m², SMD = 0.108), diabetes mellitus (54.36% vs 45.30%, SMD = 0.187), heart failure (37.48% vs 9.01%), LVEF (50.30 ± 15.10% vs 49.69 ± 19.86%, SMD < 0.001), LAVI (34.68 ± 14.68 vs 34.66 ± 18.88 mL/m², SMD < 0.001), and CHA₂DS₂-VA score (median 2 vs 3, SMD = 0.227) (Table 2).

**Table 2:** Clinical outcomes after propensity score weighting This table shows weighted incidence rates and HRs for AHREs and MACE, including MI, stroke, heart failure hospitalization, and cardiovascular death, between the SGLT2i and non-SGLT2i groups. Weighted HRs were derived from Cox proportional hazards models after propensity score overlap weighting, with estimates pooled across 20 multiply imputed datasets using Rubin’s rules. Incidence rates are expressed per 1,000 person-years. AHREs, atrial high-rate episodes; CVD, cardiovascular death; HFH, heart failure hospitalization; MACE, major adverse cardiovascular events (composite of MI, stroke, heart failure hospitalization, and cardiovascular death); MI, myocardial infarction; SGLT2i, sodium-glucose cotransporter-2 inhibitor

Outcome	SGLT2i (n = 15)			Non-SGLT2i (n = 108)			
	Number of events	Follow-up time (person-years)	Weighted incidence rate (events per 1,000 person-years, 95% CI)	Number of events	Follow-up time (person-years)	Weighted incidence rate (events per 1,000 person-years, 95% CI)	Weighted HR (95% CI)
AHREs	3	73.60	40.76 (-5.3 to 86.89)	54	41.38	1304.97 (956.91 to 1,653.04)	0.32 (0.10 to 0.98)
MACE	3	74.22	40.42 (-5.32 to 86.16)	20	82.81	241.52 (135.67 to 347.37)	1.10 (0.25 to 4.96)
MI	1	74.22	13.47 (-12.93 to 39.88)	5	82.81	60.38 (7.45 to 113.31)	8.84 (0.36 to 215.06)
Stroke	1	74.22	13.47 (-12.93 to 39.88)	5	82.81	60.38 (7.45 to 113.31)	1.43 (0.40 to 5.15)
HFH	2	74.22	26.95 (-10.40 to 64.29)	11	82.81	132.84 (54.34 to 211.34)	0.91 (0.17 to 4.90)
CVD	1	74.22	13.47 (-12.93 to 39.88)	3	82.81	36.23 (-4.77 to 77.22)	0.72 (0.81 to 6.51)

Device-related characteristics were similar between the SGLT2i and non-SGLT2i groups (Table 3).

**Table 3:** CIED parameters This table compares device type, lead positions, and percentages of atrial and ventricular pacing between the SGLT2i and non-SGLT2i groups. CIED, cardiac implantable electronic device; CRT-D, cardiac resynchronization therapy with defibrillator; CRT-P, cardiac resynchronization therapy with pacemaker; DDR, dual-chamber pacemaker, rate-responsive; HBP, His bundle pacing; LV, left ventricle; MCV, mid-lateral coronary vein (LV lead position); RV, right ventricle; RVOT, right ventricular outflow tract; SGLT2i, sodium-glucose cotransporter-2 inhibitor

Parameters	SGLT2 (n = 15)	Non-SGLT2 (n = 108)
CIED type		
DDR (n, %)	9 (60.00%)	104 (96.30%)
CRT-D (n, %)	6 (40.00%)	3 (2.78%)
CRT-P (n, %)	0 (0.00%)	1 (0.93%)
RV lead position		
Apex (n, %)	9 (60.00%)	64 (59.26%)
Septum (n, %)	6 (40.00%)	43 (39.81%)
RVOT (n, %)	0 (0.00%)	0 (0.00%)
HBP (n, %)	0 (0.00%)	1 (0.93%)
LV lead position		
MCV (n, %)	3 (20.00%)	1 (0.93%)
Anterior (n, %)	0 (0.00%)	0 (0.00%)
Posterior (n, %)	3 (20.000%)	3 (2.78%)
% A-pace (median, IQR)	31 (6.2, 60)	29.95 (7.15, 74.5)
% V-pace		
RV (median, IQR)	96.3 (1.11, 99)	27.5 (1, 99)
LV (median, IQR)	98 (97.9, 99)	97.15 (94.65, 99)

The majority of patients in both groups had dual-chamber pacemakers, while a higher proportion of SGLT2i users received CRT-D devices (40.0% vs 2.8%). Median percentages of atrial and ventricular pacing were similar between groups.

During follow-up of 73.60 person-years in SGLT2i users and 41.38 person-years in nonusers, three AHRE events occurred in SGLT2i users compared with 54 events in nonusers. The weighted incidence rate of AHREs was markedly lower in SGLT2i users at 40.76 per 1,000 person-years (95% CI: -5.36 to 86.89) versus 1,304.97 per 1,000 person-years (95% CI: 956.91-1,653.04) in nonusers. Weighted Cox proportional hazards analysis demonstrated that SGLT2i use was associated with a 68% reduction in AHRE risk (weighted HR 0.32, 95% CI 0.10-0.98, p = 0.03). Kaplan-Meier survival analysis confirmed significantly higher AHRE-free survival in the SGLT2i group (log-rank p = 0.03), with curves showing early separation maintained throughout follow-up. Among patients who developed AHREs, the mean longest single episode duration was substantially shorter in SGLT2i users compared to nonusers (0.10 hours vs 50.60 hours, p = 0.222), although this difference did not reach statistical significance.

**Figure 1:**
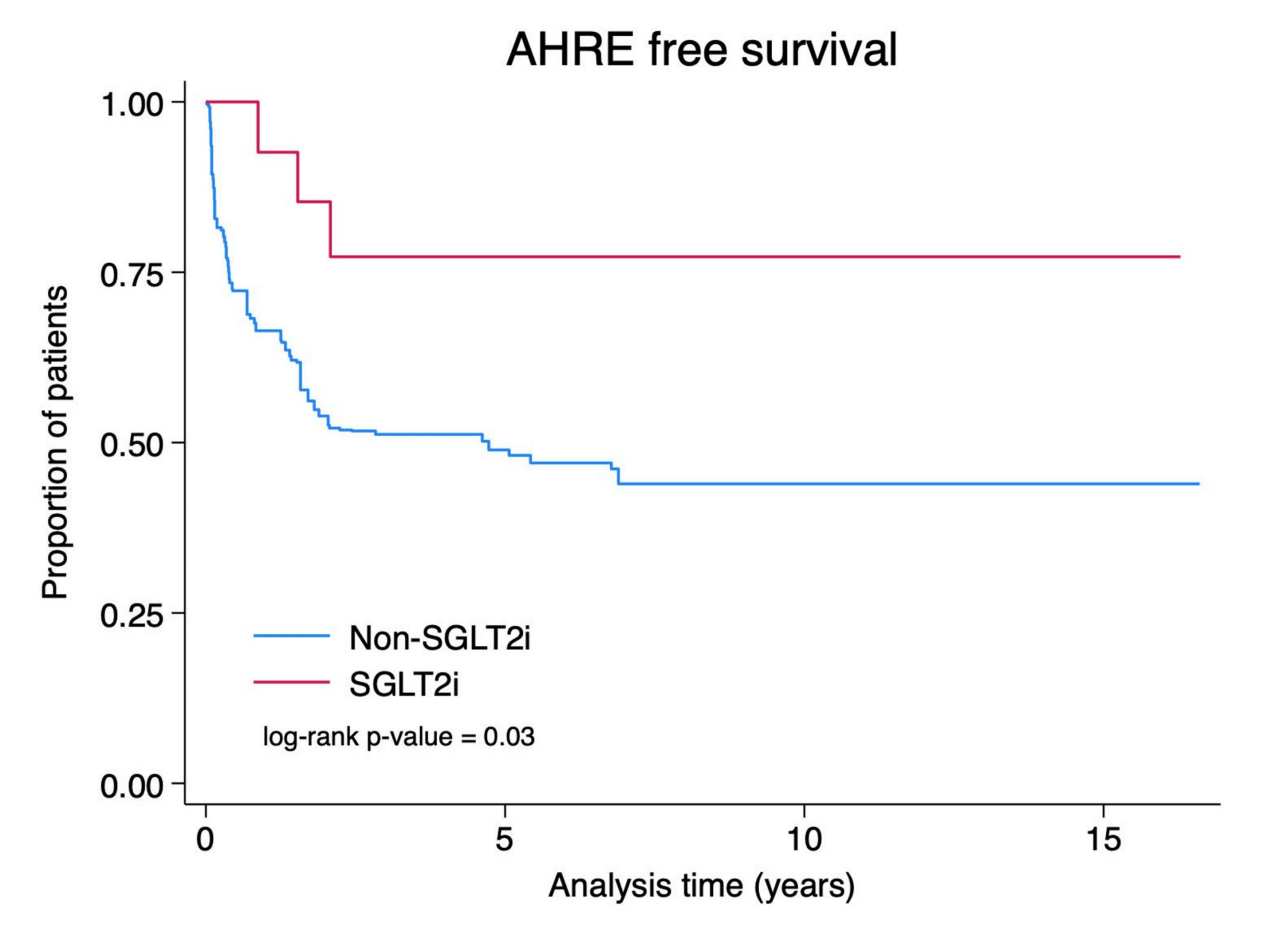
Kaplan-Meier curve for AHRE-free survival Kaplan-Meier survival analysis demonstrates significantly higher AHRE-free survival among patients receiving SGLT2i (SGLT2i group) compared with those not receiving SGLT2i (non-SGLT2i group) (log-rank p = 0.03). AHREs, atrial high-rate episodes; SGLT2i, sodium-glucose cotransporter-2 inhibitor

During follow-up of 74.22 person-years in SGLT2i users and 82.81 person-years in nonusers, MACE occurred in three patients (20.0%) in the SGLT2i group versus 20 patients (18.5%) in the non-SGLT2i group. The weighted incidence rates were 40.42 per 1,000 person-years (95% CI: -5.32 to 86.16) and 241.52 per 1,000 person-years (95% CI: 135.67-347.37), respectively. Weighted Cox regression analysis revealed no significant difference in MACE risk between groups (weighted HR 1.10, 95% CI 0.25-4.96). Analysis of individual MACE components showed the following weighted HRs for SGLT2i users versus nonusers: MI HR 8.84 (95% CI 0.36-215.06), stroke HR 1.43 (95% CI 0.40-5.15), heart failure hospitalization HR 0.91 (95% CI 0.17-4.90), and cardiovascular death HR 0.72 (95% CI 0.81-6.51). None of these associations reached statistical significance, reflecting limited statistical power due to low event rates (Figure 1).

## Discussion

This retrospective cohort study provides novel evidence that SGLT2i use is associated with a significantly reduced incidence of AHREs in patients with CIEDs. After adjustment for baseline differences, SGLT2i users demonstrated a 68% lower risk of developing AHREs compared to nonusers (HR 0.32, 95% CI 0.10-0.98, p = 0.03). These findings suggest potential antiarrhythmic benefits of SGLT2i that extend beyond their established cardiovascular protective effects.

Our results align with and extend previous research on SGLT2i and atrial arrhythmias. Zelniker et al. reported that dapagliflozin reduced clinical AF or atrial flutter by 19% in patients with type 2 diabetes in the DECLARE-TIMI 58 trial [22]; however, that study focused on clinically diagnosed AF rather than device-detected AHREs. Our study specifically examined subclinical AHREs, which often precede clinical AF and carry independent prognostic significance. A recent randomized controlled trial by Nantsupawat et al. investigated dapagliflozin’s effects on AHREs in 54 CIED patients over three months [23]. While they observed a numerical reduction in AHRE frequency (a decrease of 2.2 episodes/month in the dapagliflozin group vs an increase of 0.6 episodes/month in the placebo), the difference did not reach statistical significance, likely due to the small sample size and short follow-up. Vecchi et al. recently reported that SGLT2i reduced arrhythmic burden, including AHREs, in heart failure patients with ICDs [24]. Our study corroborates these findings in a broader CIED population with diverse indications.

Several mechanisms may explain the observed association between SGLT2i use and reduced AHRE incidence. First, SGLT2i promote osmotic diuresis and natriuresis, which may reduce intravascular volume and ventricular filling pressures. Modest afterload reduction may further improve ventricular unloading. Together, these effects can lower left ventricular diastolic pressure and pulmonary venous congestion, reducing left atrial pressure and wall stress, both important contributors to atrial stretch, remodeling, and arrhythmogenesis [13,14,18,25]. Second, SGLT2i exhibit anti-inflammatory properties, reducing markers such as C-reactive protein and cytokines [18], which may mitigate atrial fibrosis and arrhythmia substrate. Third, SGLT2i may modulate sodium-hydrogen exchanger (NHE) activity in cardiomyocytes, preventing intracellular sodium and calcium overload that can trigger arrhythmias [19,20]. Fourth, by improving glycemic control and promoting modest weight loss (2-4 kg over 6-12 months) [18,25], SGLT2i address multiple metabolic risk factors for AF.

The substantially shorter AHRE duration observed in SGLT2i users, although not statistically significant, suggests that SGLT2i may not only prevent AHRE occurrence but also limit episode duration. This observation warrants further investigation, as AHRE burden correlates with stroke risk and progression to persistent AF [5,26]. Current guidelines recommend considering anticoagulation for patients with device-detected AHREs ≥24 hours and CHA₂DS₂-VA score ≥2 [27]. If SGLT2i reduce AHRE burden below clinically significant thresholds, they may potentially decrease the need for anticoagulation and reduce associated bleeding risks.

We observed no significant difference in MACE incidence between SGLT2i users and nonusers (HR 1.10, 95% CI 0.25-4.96). This contrasts with large randomized trials demonstrating cardiovascular benefits of SGLT2i [16,19]. Several factors may explain this discrepancy. First, with only 23 total MACE events, the study was underpowered to detect differences. Second, the time from SGLT2i initiation to outcome assessment varied, potentially diluting treatment effects. Third, our CIED population may differ from typical SGLT2i trial populations in baseline cardiovascular risk and comorbidities. Individual MACE components showed wide CIs, though the point estimate for heart failure hospitalization (HR 0.91) suggested a trend consistent with established SGLT2i effects.

These findings have several clinical implications. First, SGLT2i may represent a novel pharmacological approach to preventing subclinical atrial arrhythmias in CIED patients, potentially reducing progression to clinical AF. Second, patients with CIEDs at high risk for AHREs (e.g., those with heart failure, diabetes, or prior brief AHRE episodes) may be important targets for future studies. Third, beyond glucose control and heart failure management, SGLT2i may provide additional rhythm control benefits, supporting their use as foundational therapy in appropriate patients.

The novelty of this study lies in its evaluation of device-detected, subclinical AHREs rather than clinically overt AF in a real-world CIED population undergoing routine follow-up. Additionally, we applied multiple imputation and propensity score overlap weighting to address missing data and reduce measured confounding, providing an early signal that SGLT2i therapy may be associated with a lower burden of subclinical atrial arrhythmias.

However, the observational design, small sample size, and heterogeneity in device type necessitate cautious interpretation, and findings should be considered hypothesis-generating. Larger prospective studies are needed to determine whether SGLT2i reduce subclinical atrial arrhythmia burden and influence progression to clinically overt AF.

This study has several limitations. First, despite propensity score weighting, unmeasured confounding remains possible, including residual confounding by indication. Second, the small number of SGLT2i users (n = 15) limited statistical power, particularly for subgroup analyses and MACE assessment; wide CIs reflect this imprecision. Sex-stratified analyses were not feasible due to the limited sample size. Third, although multiple imputation addressed missing LVEF and LAVI data, this introduces additional uncertainty. Fourth, findings from a single-center Thai population may not generalize to other ethnicities or healthcare settings. Fifth, retrospective outcome ascertainment may have missed events or misclassified outcomes, introducing information bias. Sixth, despite achieving good covariate balance (SMD <0.23), residual confounding from unmeasured variables (e.g., frailty status, medication adherence, and access to care) may persist. Seventh, AHRE detection and arrhythmic risk may differ by device type; the higher proportion of CRT-D devices among SGLT2i users could have influenced detection sensitivity and outcomes. Finally, the relatively short mean AHRE duration observed in SGLT2i users was based on only three events, rendering this finding exploratory and requiring confirmation in larger studies.

## Conclusions

In this retrospective cohort study of 123 patients with CIEDs, SGLT2i use was associated with a statistically significant 68% reduction in the incidence of AHREs. The weighted incidence rate of AHREs was markedly lower in SGLT2i users (40.76 per 1,000 person-years) compared to nonusers (1,304.97 per 1,000 person-years), and Kaplan-Meier analysis demonstrated superior AHRE-free survival in the SGLT2i group throughout follow-up. These findings suggest that SGLT2i may confer antiarrhythmic benefits beyond their established cardiovascular protective effects, potentially through mechanisms including attenuation of atrial remodeling, reduction of inflammation and oxidative stress, and modulation of cardiac ion channel function. Our results support the rationale for prospective randomized controlled trials to definitively evaluate the efficacy of SGLT2i in preventing AHREs and their progression to clinical AF in patients with CIEDs. If confirmed in adequately powered prospective studies, SGLT2i could represent a novel therapeutic strategy for rhythm management in this population.
